# Diagnosis Analysis of 4 TCM Patterns in Suboptimal Health Status: A Structural Equation Modelling Approach

**DOI:** 10.1155/2012/970985

**Published:** 2012-04-10

**Authors:** Li-Min Wang, Xin Zhao, Xi-Ling Wu, Yang Li, Dan-Hui Yi, Hua-Ting Cui, Jia-Xu Chen

**Affiliations:** ^1^School of Preclinical Medicine, Beijing University of Chinese Medicine, No. 11, Beisanhuan Donglu, Chaoyang District, Beijing 100029, China; ^2^Center for Applied Statistics, Renmin University of China, 59 Zhongguancun Avenue, Haidian District, Beijing 100872, China; ^3^School of Statistics, Renmin University of China, 59 Zhongguancun Avenue, Haidian District, Beijing 100872, China; ^4^School of Public Health, Yale University, 60 College Street, New Haven, CT 06511, USA; ^5^Department of Basic Theory in Chinese Medicine, Henan University of Traditional Chinese Medicine, Zhengzhou 450008, China

## Abstract

*Background*. We illustrated an example of structure equation modelling (SEM) in the research on SHS to explore the diagnosis of the Sub optimal health status (SHS) and provide evidence for the standardization of traditional Chinese medicine (TCM) patterns in SHS. And the diagnosis of 4 TCM patterns in SHS was evaluated in this analysis. *Methods*. This study assessed data on 2807 adults (aged 18 to 49) with SHS from 6 clinical centres. SEM was used to analyze the patterns of SHS in TCM. Parameters in the introduced model were estimated by the maximum likelihood method. *Results*. The discussed model fits the SHS data well with CFI = 0.851 and RMSEA = 0.075. The direct effect of Qi deficiency pattern on dampness pattern had the highest magnitude (value of estimate is 0.822). With regard to the construct of “Qi deficiency pattern”, “fire pattern”, “stagnation pattern” and “dampness pattern”, the indicators with the highest load were myasthenia of limbs, vexation, deprementia, and dizziness, respectively. It had been shown that estimate factor should indicate the important degree of different symptoms in pattern. *Conclusions*. The weights of symptoms in the respective pattern can be statistical significant and theoretical meaningful for the 4 TCM patterns identification in SHS research. The study contributed to a theoretical framework, which has implications for the diagnosis points of SHS.

## 1. Introduction

Suboptimal health state (SHS) is a physical state between health and disease and is characterized by the perception of health complaints, general weakness, and low energy [1]. In the related discussion, it is shown as energy reduction, symptoms of function, and adaptability diminishing but has not met the current diagnostic criteria for disease yet. From the view of TCM theory, *Yin* (things associate with the physical form of an object and have less energetic qualities) and *Yang* (things associate with energetic qualities), *Qi* (*Qi *is life-force, which animates the forms of the world) and *Xue* (*Xue* is a dense form of body fluids that have been acted upon and energized by *Qi*), and *Zang* (*Zang* consists of the heart including the pericardium, lung, spleen, liver, and kidney; *Zang* organs mainly manufacture and store essence: *qi*, blood, and body fluid) and *Fu* (*Fu* consists of gall bladder, stomach, large intestine, small intestine, urinary bladder, and the *Sanjiao* (three areas of the body cavity); Fu organs mainly receive and digest food, absorb nutrient substances, and transmit and excrete wastes) are in an unbalanced state though no any organic pathological changes have been found in the body when people have a subhealth state.

Recent years, SHS has become a new public health challenge all over China. The number of people who were reported suboptimal health in the absence of a diagnosable condition increased [2]. Research on classification and standardization of patterns of suboptimal health status is a hot topic in recent years [3–5]. Unfortunately, the quantitative analyses about the symptoms in different patterns of SHS in TCM are limited. However, with increasing economic development, the prevalence of SHS is expected to escalate. Studies on intervention and prognosis for SHS are expected to become increasingly important, especially in TCM clinical research. Consequently, the existence of a pattern differentiation to assess SHS will be essential. Therefore, the present study was based on the multicentral large sample clinical epidemiological investigation, and Structural Equation Model (SEM) was used to make analysis on the patterns of SHS.

## 2. Methods

### 2.1. Clinical Data Collection

The participants were cluster sampled from six clinical centres participating in this project. The centres are the Beijing Guanghua Hospital Medical Center in Beijing (BJ for short), the Hanzhong People's Hospital Medical Center in Shanxi Province (SX for short), The Hospital affiliated to Changchun University of Chinese Medicine Medical Center in Jilin Province (JL for short), the Shenzhen Second People's Hospital in Guangdong Province (GD for short), the Zhenjiang People's Hospital Medical Center in Jiangsu Province (JS for short), and the Huangshi Aikang Hospital in Hubei Province (HB for short).

The participants from the 6 clinical centres, which were sampled from over 1 million people, consisted of 2807 sub-health samples, in which 1286 were male (45.81% of the total number of cases, age 31.07 ± 0.235 years), and 1521 were female (54.19% of the total number of cases, age 32.26 ± 0.213 years). As shown in Table 1, further information on the samples was provided. Ethical approval for the research protocol and written informed consent were obtained from the ethics committee prior to the study initiation. Written informed consent was obtained from all of respondents. Self-administered questionnaire has good reliability, and validity [6, 7]. Data were collected during October 2009–March 2010.

### 2.2. Diagnostic Criteria of SHS Include the Following Two Items

More than three-month recurring illness state and efficiency decline because of persistent or excessive fatigue; and no major organic diseases and physiological or mental diseases. Case which must strictly meet the previous two criteria should be diagnosed as SHS.

### 2.3. Inclusion Criteria of SHS Also Include the Three Items as Follows

Each case must accord with the SHS diagnostic criteria; age should be from 18 through 49 years; each case must be attached with an informed consent form (ICF) signed by the respondent. Case which must all be consistent with the previous 3 items can be concluded in.

### 2.4. Additionally, Exclusion Criteria of Sub-Health State Have Five Items

Any case who do not accord with inclusion criteria; Women who are pregnant, breast-feeding, or intend to pregnant; any case who do not sign an informed consent form; any case whose questionnaire [6, 7] is incomplete filled (the absence and omitting of self-administered items except general information should not beyond 5% or no interview); and any patient who catches metabolic syndrome. Any case which meets the previous items must be excluded.

Consecutive samples with a single center are used in present study. In other words, the participants who met the inclusion criterion while not being rejected for exclusion criterion were all included, for inducing selection bias. Clinical investigators were trained so that they were fully understood the epidemiological survey programs and standard operating procedures. Epidata 3.02 was used to verify the data parallel double-inputted.

### 2.5. Statistical Analysis

**Figure 1:**
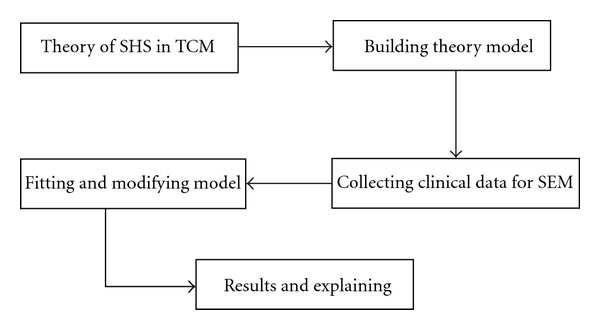
Flow chart for building SEM of SHS.

A basic structure equation model consists of two components: the measurement model which describes how indicator variables related to the latent variables and the structural model which analyzes the relationships among latent variables. The models proposed were estimated using the AMOS 16.0 program. Confirmatory factor analysis (CFA) was used to construct the measurement model structural mode, by maximum likelihood method to estimate parameters. Goodness of fit for our model was two indices of practical fit: the comparative fit indices (CFIs) and the root mean square error of approximation (RMSEA), which were in wide use and known to be relatively unaffected by sample size [8]. The model is well fitted for RMSEA being equal and less than 0.05, middle matched for RMSEA being greater than 0.08 and less than 0.1, and unmatched for RMSEA being greater than 0.1. The value of CFI is between 0 and 1. The value is bigger while model fits better [9].  Figure 1 showed the flow chart for building structure equation model of SHS.

### 2.6. Theoretical Model

Based on results of the summary research and the experts' counselling, we build the theoretical model for the basic patterns of sub-health state [10–13] and the understanding of patterns transfer regulation. The liver governs free coursing, which refers to liver *qi*'s physiological function of ensuring smooth free flow (of *qi* and Blood), so the dysfunction may lead to Stagnation pattern of liver. And long-term stagnation causes the heat; that is, the stagnation of live-*qi* can lead to the fire pattern of liver. Deficiency of spleen *qi* causes the dysfunction in water transportation and then results in Dampness pattern. The dampness obstructing long-term can cause heat and fire, so dampness pattern can lead to fire pattern. Dampness hampering *qi* movement can lead to stagnation pattern.

**Figure 2:**
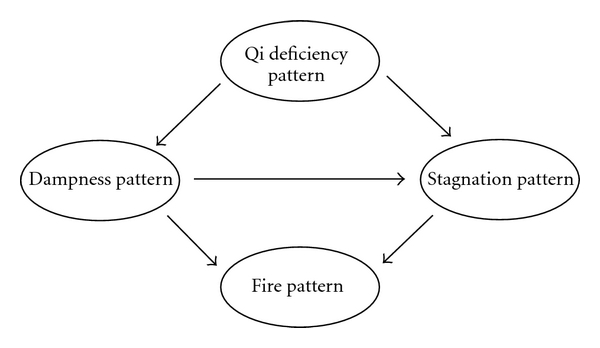
Theoretical model tested using structural equations.

Figure 2 showed the theoretical model tested. The latent variables were represented by the ellipses. The exogenous variable “Qi deficiency pattern” was composed of 6 directly observed variables, fatigue, degree of fatigue, weakness, shortness of breath, lazy speech, and dizziness. The variable “Stagnation pattern” was measured with 7 indicators, emotional depression, irritability, nervousness, anxiety, often heaving a deep sigh, hypochondriac pain, and the lower abdomen pain. The variable “fire pattern” was a latent variable with 6 indicators, bitter taste in mouth, dry pharynx, upset, deep-colored urine, constipation, and swollen sore throat. Four directly observed variables, including dizziness, sticky mouth, limpness, and drainage difficulty, were used to construct the latent variable “dampness pattern”.

## 3. Results

### 3.1. Measurement Model

The first step in the structural equation analysis was the construction of the measurement model. The initial measurement model was constructed on the understanding of patterns transfer regulation in SHS. The factor loadings of the indicators of the latent construct “Qi deficiency pattern” were all higher than 0.60, the two inverse items (x12 and x02) excepted. The indicator with the highest load for this construct was myasthenia of limbs. This indicates that the latent variable adequately predicted the variability of the observed variable (Figure 2). With regard to the constructs “fire pattern”, “stagnation pattern”, and “dampness pattern”, the indicators with the highest load were vexation, deprementia, and dizziness, respectively. In the main symptoms of stagnation pattern, the load coefficient of emotional depression and nervousness was higher than that of hypochondriac pain and lower abdomen pain. It was shown that emotional symptoms for diagnosis of stagnation pattern have greater weight. That was different from the other stagnation patterns of disease status; hypochondriac pain and lower abdomen pain had the greater weight [14, 15].

### 3.2. Structure Model

Standardized coefficients of the structural model obtained for the SHS were presented in Table 2. These coefficients indicated the impact on the response variable relative to the variation of one standard deviation unit in the explanatory variable. The direct effect of Qi deficiency pattern on dampness pattern was of the highest magnitude (value of estimate is 0.822), and then on the stagnation pattern (value of estimate is 0.351). This implied that for each variation of one standard deviation in Qi deficiency pattern there was a significant increase of 0.822 standard deviation in dampness pattern.

**Figure 3:**
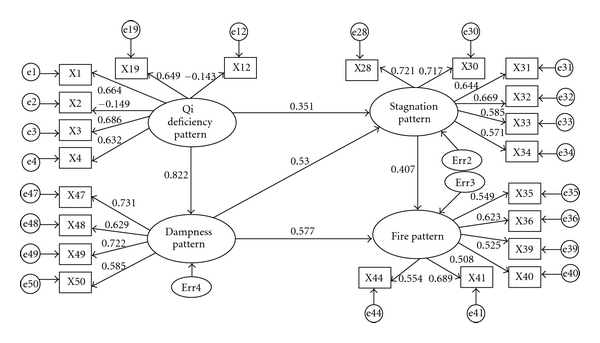
Structural equation model of SHS.

 In the same way, the direct effect of Qi deficiency pattern on myasthenia of limbs was of the highest magnitude (value of estimate is 0.686), and then on fatigue (value of estimate is 0.664). This implied that for each variation of one standard deviation in Qi deficiency pattern there was a significant increase of 0.686 standard deviation in myasthenia of limbs and of 0.664 standard deviation in fatigue. The fit of our model provided a middle fit to our data with CFI = 0.851 and RMSEA = 0.075. All of the paths in the final model were highly significant. The final model was represented in Figure 3 and the factor loadings of the measurement model were shown in Table 3.

## 4. Discussion

TCM pattern is a generalization of various symptoms and signs occurring in a certain stage of a disease, investigating causes, pathogenesis, pathological manifestation, location, and nature of disease. Pattern is an abstraction idea based on the symptoms or signs. It is similar to latent variable which should be quantified and made objective. Pattern identification is a method of thinking which provides evidence for treatment by synthesizing and analyzing clinical data and differentiating patterns on the basis of TCM theories. 

Structural equation modelling integrates the idea of factor analysis, correlation analysis, and regression analysis. It can inference on the direct and indirect effects among variables [16–18] besides the analysis of the observation latent variables and measurable variables. With data mining technology widely used in TCM diagnosis [19] and clinical research, SEM was also applied in the study of TCM syndrome standards [18–21]. 

The results of this study indicate that the SHS model provided middle fit to the data obtained from a large cross-sectional clinical epidemiological investigation. It would be helpful to know for both clinical and research purposes, for example, which variable (symptom) is important to the SHS pattern identification. 

Our findings were consistent with the theory of TCM pattern. Effects of Qi deficiency pattern on dampness pattern (0.822) were greater than those on stagnation pattern (0.351). The fact of Qi deficiency of spleen leading to dampness pattern was more obvious than the fact of Qi deficiency of liver leading to stagnation pattern, which was related to the fact of Qi deficiency of spleen being more popular than Qi deficiency of liver and consistent with the fact of liver stagnation and Qi deficiency of spleen pattern being the popular pattern of SHS [10]. Effects of dampness pattern on fire pattern (0.577) were greater than those on stagnation pattern (0.520). It is shown that the dampness obstructing long-term can cause heat and fire. Further, effects of stagnation pattern on fire pattern (0.407) were less than those of stagnation pattern on fire pattern (0.577). It was probably due to effects of Qi deficiency pattern on dampness pattern being greater than those on stagnation pattern, which had indirect effect on the degree of influence of dampness pattern and stagnation pattern on fire pattern. 

Furthermore, to a certain degree, the study presented here revealed that the weights of symptoms in the respective pattern represent importance to the pattern identification in SHS. The symptoms of different patterns showed the specific standardized factor loadings, which indicate the weights in their respective patterns and the exact diagnosis of patterns. The exogenous variable “Qi deficiency pattern” was composed of 6 directly observed variables, fatigue, degree of fatigue, weakness, shortness of breath, lazy speech, and dizziness. The variable “stagnation pattern” was measured with 7 indicators, emotional depression, irritability, nervousness, anxiety, often heaving a deep sigh, hypochondriac pain, and the lower abdomen pain. In the main symptoms of stagnation pattern, the load coefficient of emotional depression and nervousness was higher than that of hypochondriac pain and lower abdomen pain. It was shown that emotional symptoms for diagnosis of stagnation pattern had greater weight. That was different from the other stagnation patterns of diseases; hypochondriac pain and lower abdomen pain had the greater weight [14, 15]. In general, the weights of symptoms in the respective pattern can be significant for 4 TCM patterns identification in SHS. 

One of the limitations of this study was that all variables were assessed using questionnaires [6, 7]; results may have been biased by the common method variance. This level of bias was a real cause for concern in survey studies because the common method variance may enhance the observed correlation between variables [22]. Another limitation in our present study was the rejection of subpatterns related to Qi deficiency pattern and fire pattern, which should have a certain influence to thoroughly analyze SHS patterns. Despite the afore mentioned limitations, the overall findings of the study suggested that the use of SEM enables us to find and support the possible cause-effect relationship between latent variables (patterns) and measurable variables (symptoms) in SHS. Therefore, by using SEM analysis, we can provide establishing of diagnostic criteria patterns of SHS. In future studies, it would therefore be valuable to test the quantification diagnosis of SHS subpatterns within the clinical setting. 

## 5. Conclusions

In conclusion, we have demonstrated that the use of SEM enables us to find and support the impossible cause-effect relationship between latent variables (patterns) and measurable variables (symptoms) in SHS. The study contributed to a theoretical framework, which had implications for the diagnosis points of SHS. To a certain degree, the weights of symptoms in the respective pattern represented importance to the pattern identification in SHS. It was shown that emotional symptoms for diagnosis of stagnation pattern have greater weight in SHS.

## Figures and Tables

**Table 1:** Characteristics of the samples in different areas.

	BJ	SX	JL	GD	JS	HB
Sample size	717	452	463	486	563	666
No(%) of sub	564 (78.7%)	418 (58.3%)	448 (62.5%)	431(60.1%)	445 (62.1%)	501 (69.9%)
Mean age (SD) of sub	30.41 ± 0.298	33.19 ± 0.39.	34.13 ± 0.389	30.72 ± 0.369	33.81 ± 0.446	28.78 ± 0.298

**Table 2:** The standardized coefficients of the structural model.

Effects		Estimate
Y4 dampness syndrome	←Y1 Qi deficiency pattern	.822
Y3 fire syndrome	←Y4 dampness pattern	.577
Y2 stagnation syndrome	←Y4 dampness pattern	.520
Y3 fire syndrome	←Y2 stagnation pattern	.407
Y2 Stagnation syndrome	←Y1 Qi deficiency pattern	.351

**Table 3:** Shows the factor loadings of the measurement model.

Effects		Estimate
x03 myasthenia of limbs	←Y1 Qi deficiency pattern	0.686
x01 fatigue	←Y1 Qi deficiency pattern	0.664
x19 disinclination to say	←Y1 Qi deficiency pattern	0.649
x04 short breath	←Y1 Qi deficiency pattern	0.632
x12 inferiority	←Y1 Qi deficiency pattern	−0.143
x02 degree of fatigue	←Y1 Qi deficiency pattern	−0.149
x41 vexation	←Y3 fire pattern	0.689
x36 dry pharynx	←Y3 fire pattern	0.623
x44 swollen sore throat	←Y3 fire pattern	0.554
x35 bitter taste of mouth	←Y3 fire pattern	0.549
x39 constipation	←Y3 fire pattern	0.525
x40 deep-colored urine	←Y3 fire pattern	0.508
x28 deprementia	←Y2 stagnation pattern	0.721
x30 nervous	←Y2 stagnation pattern	0.717
x32 be apt to breathe	←Y2 stagnation pattern	0.669
x31 anxiety	←Y2 stagnation pattern	0.644
x33 hypochondriac distension and pain	←Y2 stagnation pattern	0.585
x34 abdominal distension and pain	←Y2 stagnation pattern	0.571
x47 dizziness	←Y4 dampness pattern	0.731
x49 limpness	←Y4 dampness pattern	0.722
x48 sticky mouth	←Y4 dampness pattern	0.629
x50 drainage difficulty	←Y4 dampness pattern	0.585
